# Prevalence and associated factors of burnout among Debre Berhan University medical students: a cross-sectional study

**DOI:** 10.1186/s12909-019-1864-8

**Published:** 2019-11-08

**Authors:** Yohannes Gebreegziabhere Haile, Amanuel Lemma Senkute, Berhanu Tadesse Alemu, Dawit Mamo Bedane, Kaleab Berhanu Kebede

**Affiliations:** 1 Department of Nursing, Debre Berhan University, Debre Berhan, Ethiopia; 2 Department of Medicine, Bench Magi Hospital, Bench Magi, Ethiopia; 3 Department of Medicine, Arsi Asela Hospital, Asela, Ethiopia; 4 Department of Medicine, Debre Berhan University, Debre Berhan, Ethiopia

**Keywords:** Burnout, Professional burnout, Medical students, Debre Berhan University, Ethiopia

## Abstract

**Background:**

Burnout, a measure of professional distress, is more common among medical professionals. About half of medical students have this problem. However, little is known about the burnout status of medical students in Ethiopia. Therefore, the aim of this study was to assess the prevalence and associated factors of burnout among medical students of Debre Berhan University (DBU).

**Methods:**

A cross-sectional study was conducted on randomly selected 151 medical students of DBU. Burnout was assessed using the Maslach Burnout Inventory-Human Services Survey (MBI-HSS). Participants were reported as having burnout if they scored ≥27 on Emotional Exhaustion (EE), ≥13 on Depersonalization (DP) sub-scales, and ≤ 31 on Personal Accomplishment (PA) sub-scale of the MBI-HSS. EpiData version 3.1 was used for data entry while SPSS version 20 and STATA version 13 for windows were used for data analysis. Both univariable and multivariable binary logistic regression analyses were conducted. The degree of association between variables was assessed using odds ratio (OR) with 95% confidence interval (CI) at two-tailed *p*-value of < 0.05.

**Result:**

Of 144 medical students took part, 34.0% had symptoms of burnout. Regarding domains of burnout, 61.8% scored high on EE, 47.9% scored high on DP and 59.7% scored low on PA. Dissatisfaction with practice lecturer (AOR = 3.8, 95% CI (1.3, 11.6)), moderate social support (AOR = 0.2, 95% CI (0.1, 0.8)), and satisfaction with their education (AOR = 0.1 95% CI (0.0, 0.7)) were associated with burnout.

**Conclusion:**

More than one-third of medical students at DBU had burnout. Individual and organizational level interventions targeting students who had poor social support, dissatisfied by their lecturer at the hospitals and their education are recommended.

## Background

Burnout is a measure of professional distress, which is thought to have three main domains: excess emotional exhaustion (EE), excess depersonalization (DP) and reduced personal accomplishment (PA) [1]. Burnout is increasingly reported as a common phenomenon among medical students, residents, and physicians with less than 5 years of experience compared to the general population [2]. This may be due to the heightened exposure to emotional strains given that they often interact with individuals who are physically and/or psychologically impaired. The prevalence of burnout among medical students ranges between 10 and 70% [3–16]. In the United States of America (USA), more than half of medical students are affected by burnout during their medical education [17].

Burnout among medical students has many consequences. Burnout is reported to be associated with job turnover, absenteeism, low morale and job dissatisfaction [18, 19]. Burnout may also be associated with some individual-level adverse outcomes, such as; poor decision making, hostility to patients, medical errors, poor relationships with colleagues, depression, anxiety and fatigue, sleep disturbances, alcoholism, drug misuse, and suicidal ideation [4, 14, 17, 20]. In addition, an increase in level of burnout showed a significant association with poor quality of life, and hypertension and other cardiovascular disorders [21–23].

Associated factors of burnout among medical student were increased year of education, presence of multiple adverse life events, stress, feelings of having limited control over life, poor peer interactions and support from friends, lower level of physical activity, smoking cigarettes, sex and excessive alcohol drinking [3, 6, 7]. Other studies found association between burnout and lack of confidence in once clinical skills, feeling uncomfortable with course activities, falling to see coursework as a source of pleasure, falling an examination, considering withdrawal from school and increased age. Moreover, having parents who are doctors, lack of supportive resources, having shortage of off-time, lack of belief in the importance of their work, fear of the consequences of failure, having family responsibilities, having an uncertain future, having a stress, and having increased workload are also reported to be associated with increased burnout level [8–10, 16].

In Ethiopia, very few studies were conducted on burnout and none of them were on medical students. These studies focuses on conceptualization of burnout among primary health workers [24], assessing factors that affect the motivation of health care workers [25], and burnout status and work-related stress of the health professionals working at hospitals [26, 27]. Medical students in Ethiopia are at a high stressful situation because of economic problems, which may lead to burnout. At Debre Berhan University (DBU), most medical students had added family responsibilities in addition to their education that may lead to burnout. In addition, medical students of DBU are recruited after having a bachelor’s degree and used to work at least for 2 years, as a result there might be role conflict when they were changed from employee to unemployed students. Those and other factors may increase the burden of burnout among DBU medical students to be higher than previously reported. Therefore, it is worthful to address this gap and manage burnout among medical students. Hence, this study aimed for assessing the prevalence and associated factors of burnout among medical students at DBU.

## Methods

### Study setting and period

This study was conducted among medical students at DBU from April 10 to 15, 2017. DBU is a public university located at Debre Berhan town, which is 130 km north of Addis Ababa, the capital city of Ethiopia. Department of medicine is one of the 33 departments at DBU, during the 2016/2017 academic year 213 medical students were enrolled.

### Study design and population

An institution-based cross-sectional study was conducted among 151 medical students. The sample size was determined by applying a single proportion formula with the assumption of 50% prevalence of burnout, 5% marginal error, 95% confidence level, and 10% of non-response rate. A prevalence of 50% was used to maximize the sample size since there was no previous study in DBU. Sample size correction formula was also applied given that the total number of students at DBU is less than 10,000.

A stratified sapling technique was employed using student’s class year as a stratum. First the total sample size (i.e., 151) was proportionally allocated for each stratum, then a systematic sampling method was used to obtain the study participants from each class year. We used the students’ attendance sheet, at each class year, as a sampling frame. All medical students at DBU who were available during the data collection period and willing to participate were included, while students who were too ill to complete the questionnaire were excluded.

### Measurement of variables in the study

#### Outcome variable

##### Burnout status

Burnout was the outcome variable. We have used the Maslach Burnout Inventory-Human Service Survey (MBIHSS) questionnaire to assess burnout. It is derived from the Maslach Burnout Inventory (MBI), designed for professionals in the human services. It is also appropriate for respondents working in a diverse array of occupations, including medical students. It contains 22 questions which ask participants to indicate the frequency of various feelings experience. Each question has a six-point response scale (0: “never”, 1: “a few times a year or less”, 2: “once a month or less”, 3: “a few times a month”, 4: “once a week”, 5: “a few times a week”, and 6: “every day”), and is designed to measure the three domains of burnout; i.e., EE, DP, and PA. EE is characterized by feelings of emotional overextension as a result of one’s work, DP is characterized by emotional indifference and the dehumanization of the recipients of one’s services, and PA is characterized by feelings of occupational stagnation, incompetence, and underachievement. Scores in each of the three subscales were categorized into high, average, or low scores according to cut-offs detailed in the MBI manual [28, 29]. High EE is indicated by a score ≥ 27, high DP is indicated by a score ≥ 13, and low PA is indicated by a score ≤ 31. Participants were identified as having a burnout if they scored high in EE and DP, and low in PA. The MBI-HSS is a reliable and valid instrument to assess burnout [28, 29]. A previous study has indicated that even when used in another language (Spanish) the instrument produced high sensitivity and specificity, 92.2 and 92.1% respectively [30].

##### Exposure variable

The exposure variables were selected based on previous studies report [3, 6–10, 16]. We categorized exposure variables into socio-demographic and economic information, academic and work-related factors, social support and level of satisfaction in life, personal life experiences, leisure time, and family-related factors, and substance use habit.

##### Socio-demographic and economic information

Socio-demographic and economic information were assessed using a self-structured questionnaire, which had 11 questions concerning the sex, age, field of study, relationship status, ethnicity, religion, and economic status of the students.

##### Academic and work-related factors

Eighteen questions were used to assess academic and work-related factors that might increase the risk of burnout. The items in this section assessed the learning environment, comfort with the teaching-learning process, relationship with seniors, perceived flexibility of the curriculum, and exposure to suffering.

##### Social support and level of satisfaction in life

Social support was measured by the Oslo 3-items social support scale. The score ranges from 3 to 14. Score of 3 to 8 indicate poor support, 9 to 11 indicate moderate support, and 12 to 14 indicate strong support. The Oslo-3 scale has been used in previous studies and confirmed as feasibile and valid tool [31–33]. Level of satisfaction in life was measured based on the worst week of the last month using a question that is rated from very poor to very good.

##### Personal life experiences, leisure time, and family-related factors

Nine questions were used to assess personal life experiences, leisure time, and family-related factors. Questions included were uncertainty about the future, family responsibilities, and perceived adequacy of the amount of off-time and satisfaction with the study and clinical skill.

##### Substance use habit

Nine questions were used to assess the frequency of use of commonly available substances around Debre Behan, such as cigarettes, alcohol, and khat (i.e., an amphetamine-like substance common in Ethiopia).

#### Data collection procedure

After obtaining informed consent, the data were collected using self-administered questionnaire in English language. Trained students and supervisors who have previous experience of facilitating data collection. Confidentiality was ensured for all the study participants throughout the data collection process. The respondents were encouraged to respond to all items in the questionnaire as completely as possible.

#### Data analysis

Double data entry using EpiData version 3.1 for Windows was performed after checking for completeness and inconsistencies. Afterwards, the data were analyzed using SPSS version 23 and Stata Version 13 software packages for Windows. A comma-delimited file (*.csv) file of cleaned raw data was available and supplemented as an additional file 1. The data were summarized using mean, standard deviation (SD), or percentage and the results were presented using tables and figures. While the instrument used in this study has previously been proven reliable and valid, we also conducted a reliability analysis for our scale. Associations between dependent and independent variables were tested using the odds ratio (OR) with 95% confidence interval (CI) in the univariable and multivariable binary logistic regression analysis. Variables with a *p*-value of ≤ 0.05 in the univariable model were included in the multivariable model. All variables with a *p*-value of < 0.05 in the multivariable model were considered as statistically significant.

## Results

### Socio-demographic and economic information

Of the 151 medical students invited, 95.3% (*n* = 144) students participated. The most common reason of non-participation was shortage of time.

As illustrated in the Table 1, 85.4% (*n* = 123) were male, 68.1% (*n* = 98) were from Amhara Ethnic background, 63.2% (*n* = 91) were single, and 27.1% (*n* = 39) were fourth year, students. In addition, 78.5% (*n* = 113) of the participants perceived that their income was not enough.

**Table 1:** Sociodemographic and Economic Information of the Study Participants

Variable	Response	Frequency (*n* = 144)	Percentage (%)
Sex	Male	123	85.4
	Female	21	14.6
Marital status	Single	91	63.2
	In relationship	23	16.0
	Married	30	20.8
Year of study	1st Year	17	11.8
	2nd Year	28	19.4
	3rd Year	28	19.4
	4th Year	39	27.1
	5th Year	32	22.2
Ethnicity	Amhara	98	68.1
	Oromo	25	17.4
	Tigray	8	5.6
	Other ^a^	13	9.0
Religion	Orthodox	121	84.0
	Muslim	11	7.6
	Protestant	12	8.3
Perception of having enough income	Yes	31	21.5
	No	113	78.5

^a^Gurage, Wolayta, Kambata, Argoba and Agew

The mean age of the participants was 30 years, (standard deviation (SD)) ± 3 years). The mean Grade Point Average (GPA) of the study participants was 2.93 points, (SD ± 0.41) out of four points. Likewise, the mean monthly income of the participants was 1903, (SD ± 1251) Ethiopian Birr (ETB), (1 USD = 22.607 ETB, during the data collection period) (Table 2).

**Table 2:** Summary Statistics of Continuous Variables from Sociodemographic and Economic Characteristics of the Study Participants

Variable	Mean	SD	Minimum	Maximum
Age (years)	29.51	2.84	24	38
GPA (points)	2.93	0.41	2.00	3.95
Monthly income (ETB)	1902.65	1251.22	100	5000

### Academic and work-related factors

Most of the participants were not satisfied with the learning environment (83.3%) or the teaching-learning process (90.3%). Eighty-six percent (*n* = 86) of the participants respond that the distance between the University and the Hospital had an impact on their clerkship program. Majority of the study participants were also not satisfied with the class (79.9%) and practical lecturers, (72.9%). In addition, nearly all (98.6%) of the study participants had study overload, 38.2% (*n* = 55) of them complained about administrative problems, and only 15.3% (*n* = 11) were comfortable during night duties, most of the time.

Regarding the academic environment, 66.7% (*n* = 96) of the study participants were bothered by the education system, 81.3% (*n* = 117) had academic pressure, and 55.6% (*n* = 80) had fair relationships with the seniors. Additionally, majority of the participant believed that the curriculum of medical school at DBU is inflexible (84.0%) and lacks enough off-time (77.8%) (Table 3).

**Table 3:** Academic and Work-related Factors of the Study Participants

Variable		Response	Frequency (*n* = 144)	Percentage (%)
Being comfortable with the learning environment		Yes	24	16.7
		No	120	83.3
Being comfortable with the teaching-learning process		Yes	14	9.7
		No	130	90.3
Impact of the distance between university and hospital on the clerkship program		Yes	86	86.0
		No	14	14.0
Satisfied with practices lecturers		Yes	39	27.1
		No	105	72.9
Satisfied with class lecturers		Yes	29	20.1
		No	115	79.9
Administrative problems		Yes^a^	55	38.2
		No	89	61.8
Bothered by the educational system		Yes	96	66.7
		No	48	33.3
Study overload		Yes	142	98.6
		No	2	1.4
Academic pressure		Yes	117	81.3
		No	27	18.8
Spending most of the time in the Hospital		Yes	68	68.0
		No	32	32.0
Exposure to human suffering		Yes	67	67.0
		No	33	33.0
Student-senior relationship		Bad	12	8.3
		Fair	80	55.6
		Good	52	36.1
The rigidness of the curriculum		very tight	121	84.0
		Fairly enough time	20	13.9
		Enough time	3	2.1
Rotation in hospital wards is stressful		Yes	57	57.0
		No	43	43.0
Stressful unit in Hospital	Emergency	Yes	46	46.0
		No	54	54.0
	Obs. and Gyn.	Yes	27	27.0
		No	73	73.0
	Internal medicine	Yes	39	39.0
		No	61	61.0
	Paediatrics	Yes	15	15.0
		No	85	85.0
	Surgery	Yes	38	38.0
		No	62	62.0
Comfortable with night duties		not at all	30	41.7
		sometimes	31	43.1
		most of the time	11	15.3
Courses and their time allocation		no enough	112	77.8
		enough time	28	19.4
		excess allocation of time	4	2.8

^a^lack of senior lecturer in some courses, lack of exam schedule, delay in problem-solving, poor coordination, transportation problem and poor attention given for their questions

### Social support and level of satisfaction in life

**Fig. 1:**
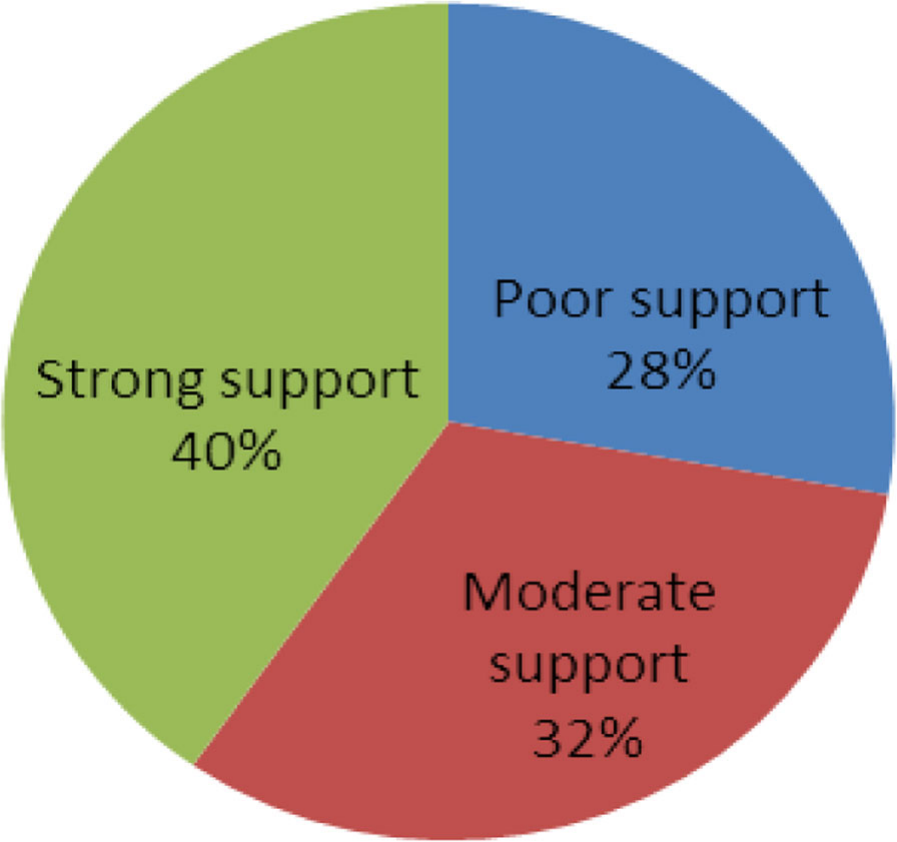
Social Support Status of the Study Participants

This study demonstrated that 40.3% (*n* = 58) of the study participants had strong social support. In the past month, nearly half (52.1%) of students had also good level life of satisfaction (Fig. 1 and Table 4).

**Table 4:** Description of Level of Overall Satisfaction of the Study Participants

Variable	Response	Frequency (*n* = 144)	Percentage (%)
Overall level of satisfaction	Very good	14	9.7
	Good	75	52.1
	Fair	47	32.6
	Poor	8	5.6

### Personal life experiences, leisure time and family-related factors

About two-third (*n* = 93) of the study participants had fair level of satisfaction with their education, 88.9% (*n* = 128) had no enough off-time for entertainment, and 63.9% (*n* = 92) afraid of failure. About two-thirds of (73.0%) the study participants had confidence in clinical skill, 77.1% (*n* = 111) had help from families, and 36.1% (*n* = 52) had added family responsibilities. Moreover, 35.4% (*n* = 51) of the study participants never perform regular exercise (Table 5).

**Table 5:** Personal, Leisure Time, and Family-Related Factors Among the Study Participants

Variable	Response	Frequency (*n* = 144)	Percentage (%)
Perceived level of satisfaction with Education	Poor	20	13.9
	Fair	93	64.6
	Good	31	21.5
Confidence in clinical skill	Yes	73	73.0
	No	27	27.0
Fear of failure	Yes	92	63.9
	No	52	36.1
Worried about future	Yes	70	48.6
	No	74	51.4
Enough off-time for entertainment	Yes	16	11.1
	No	128	88.9
Doctor family member	Yes	11	7.6
	No	133	92.4
Additional family responsibilities	Yes	52	36.1
	No	92	63.9
Help from families	Yes	111	77.1
	No	33	22.9
Practices regular exercise	Never	51	35.4
	Occasionally	90	62.5
	Almost every day	3	2.1

### Substance use habit

Concerning substance use habit, 27.8% (*n* = 40), 26.4% (*n* = 38), and 5.6% (*n* = 8) of the study participants used alcohol, khat and cigarettes, respectively. The study also showed that 25.4% (*n* = 37) of the study participants used coffee, tea, or shisha (Table 6).

**Table 6:** Description of Substance Use Habit of the Study Participants

Variable	Response	Frequency (*n* = 144)	Percentage (%)
Smoking Cigarette	Yes	8	5.6
	No	136	94.4
Chewing Khat	Yes	38	26.4
	No	106	73.6
Drinking Alcohol	Yes	40	27.8
	No	104	72.2
Use of other substance	Yes^a^	37	25.8
	No	106	74.2

^a^Coffee, Tea, and Shisha

### Reliability of MBIHSS

The internal consistency of the instrument was very high: α = 0.90 for EE subscale, α = 0.86 for DP subscale and α = 0.90 for PA subscale. In any of the sub-scales, the α for correlated item-total correlation was not less than 0.4 and removing items does not significantly improve the overall internal consistency. In addition, two-way mixed-effects model and average consistency measure were used to measure the intra-class correlation of items. In all sub-scales, the intra-class correlation were significant with an intra-class correlation of 0.90 (95% CI = 0.88, 0.92) for EE sub-scale, 0.86 (95% CI = 0.82, 0.89) for DP sub-scale, and 0.90 (95% CI = 0.88, 0.93) for PA subscale.

### Prevalence of burnout

**Fig. 2:**
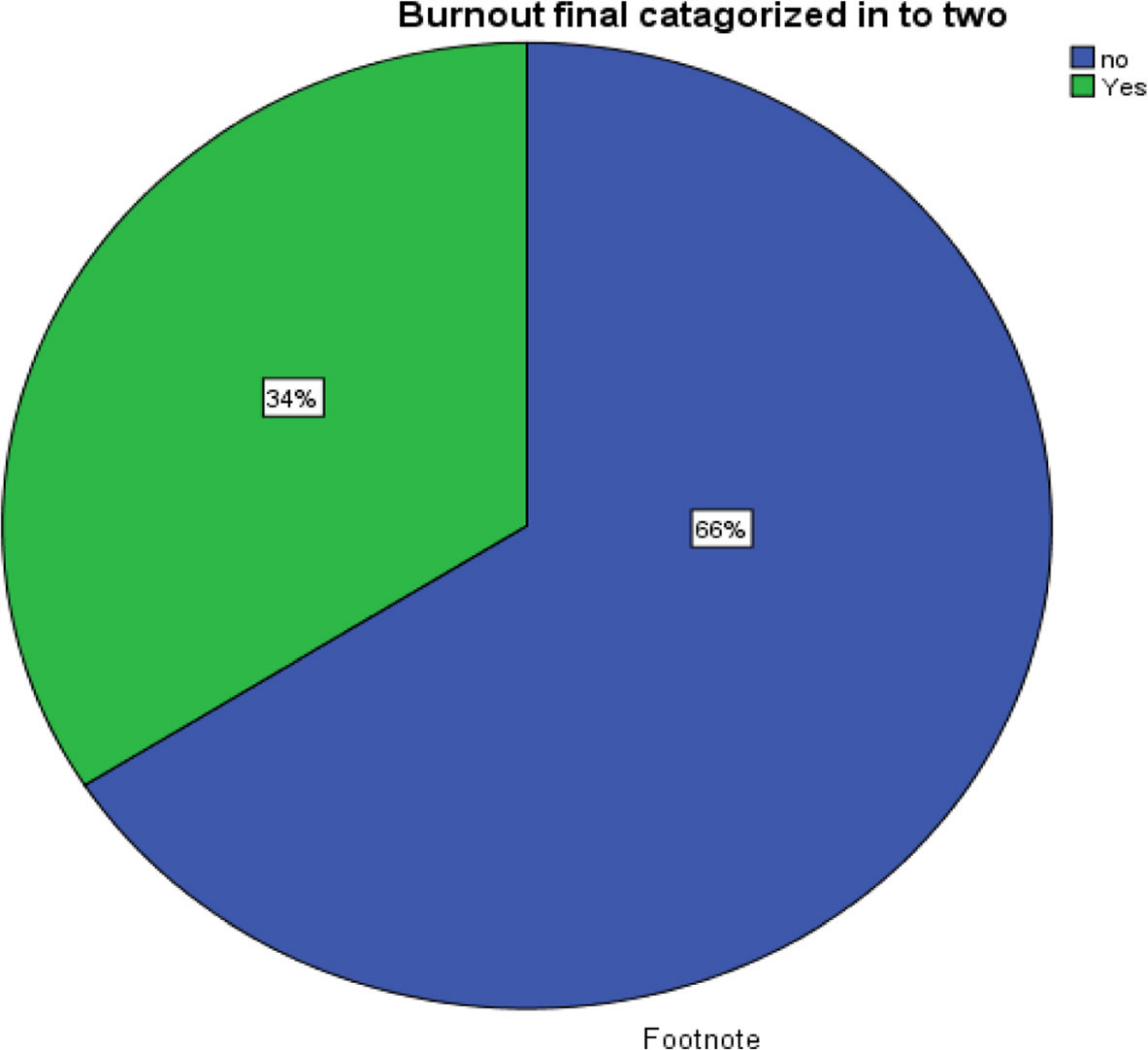
Pictorial Description of the Burnout Status of the Study Participants

**Fig. 3:**
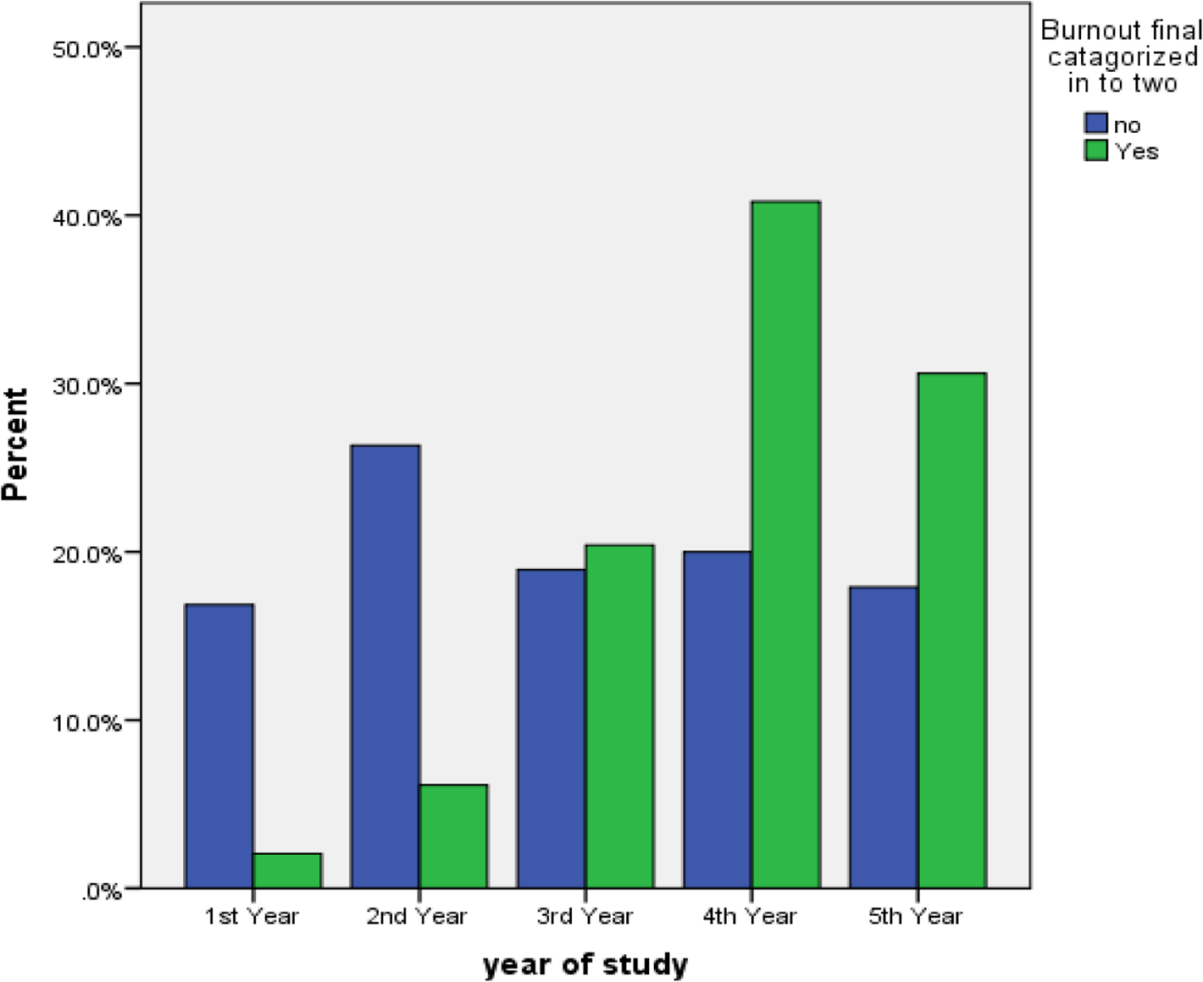
Pictorial Description of the Burnout Status by year of the Study Participants

**Fig. 4:**
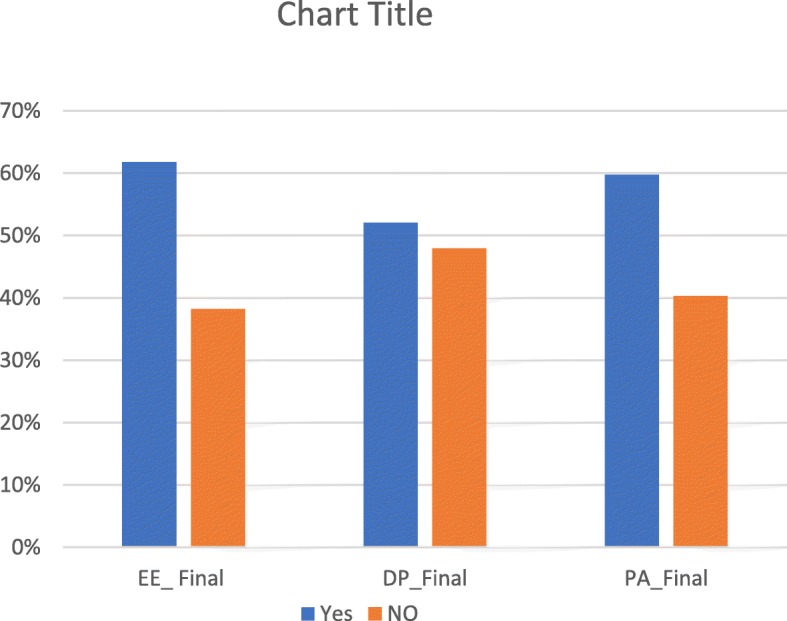
Pictorial Description of the Three subscales of MBI-HSS among the Study Participants

Based on MBI-HSS definition, 34.0% (*n* = 49) of the study participants was found to have symptoms of burnout. The burnout prevalence incrementally increased with each additional year of school until 4^th^ year and then starts to decline. Pertaining to sub-scales of MBI-HSS, 61.8% (*n* = 89) of the study participants scored high on EE sub-scale, 47.9% (*n* = 69) of the study participants scored high DP sub-scales, and 59.7% (*n* = 86) of the study participants scored low on PA sub-scale (Figs. 2, 3 and 4).

### Factors associated with burnout

Female sex, being a 2^nd^ year student, dissatisfaction with practical lecturers, not reporting administrative problem, perceiving paediatrics and emergency department as a stressful unit, being not comfortable with night duties, poor social support, very good overall life satisfaction, poor level of satisfaction on education, and not performing regular physical exercise were significantly associated with burnout in univariable model at *p*-value of < 0.05. However, perceiving paediatrics department as a stress full unit, being not comfortable with night duties, overall life satisfaction, and not performing regular physical exercise were removed from the multivariable model given that the observation per subgroup was less than five individuals.

All variables with a *p*-value of ≤0.05 in univariable model and with more than five observations per subgroup were included in the multivariable model. After controlling for the effects of potential confounding variables, dissatisfaction with practice lecturers, poor social support, and poor satisfaction on the education were significantly associated with burnout.

Students who were not satisfied by their practical lecturers were almost four times more likely to develop burnout than those who were satisfied (Adjusted Odds Ratio (AOR) = 3.8; 95% CI (1.3–11.6)). On the other hand, students who had moderate social support were 80% less likely to develop burnout than their counter parts (AOR = 0.2; 95% CI (0.1–0.8)). Additionally, students who had good levels of satisfaction on their education were 90% less likely to suffer from burnout than those who had poor satisfaction on their education (AOR = 0.1; 95% CI (0.0, 0.7)) (Table 7).

**Table 7:** Univariable and Multivariable Level Binary Logistic Regressions Analysis of Selected Variables with Burnout Status

Variable	Response	Burnout		COR (95% CI)	*p*-value	AOR (95% CI)	*p*-value
		Non-cases	cases				
Sex	Male	86 (69.9)	37 (30.1)	R		R	
	Female	9 (42.7)	12 (57.1)	3.1 (1.2, 8.0)	0.019	2.4 (0.6, 9.9)	0.215
Age	≤ 28	43 (70.5)	18 (29.5)	R		R	
	(28–31)	24 (57.1)	18 (42.7)	1.8 (0.8, 4.1)	0.165	0.5 (0.1, 1.6)	0.220
	≥ 31	28 (68.3)	13 (31.7)	1.1 (0.5, 2.6)	0.813	0.4 (0.1, 1.4)	0.139
Satisfied with practices lecturers	Yes	31 (79.5)	8 (20.5)	R		R	
	No	64 (61.0)	41 (39.0)	2.5 (1.0, 5.9)	0.041	3.8 (1.3, 11.6)	0.018*
Administrative problems	Yes*	42 (76.4)	13 (23.6)	0.5 (0.2, 1.0)	0.041	0.6 (0.2, 1.6)	0.282
	No	53 (59.6)	36 (40.4)	R		R	
Emergency department as stressful unit	Yes	20 (43.5)	26 (56.5)	2.4 (1.1, 5.4)	0.034	2.6 (1.0, 6.8)	0.060
	No	35 (64.8)	19 (35.2)	R		R	
Social support	Poor	18 (45.0)	22 (55.0)	R		R	
	Moderate	38 (82.6)	8 (17.4)	0.17 (0.1, 0.5)	< 0.0001	0.2 (0.1, 0.8)	0.019*
	Strong	39 (67.2)	19 (32.8)	0.4 (0.2, 0.9)	0.030	0.8 (0.2, 2.6)	0.692
Satisfaction on Education	Poor	9 (45.0)	11 (55.0)	R		R	
	Fair	61 (65.6)	32 (34.4)	0.4 (0.2, 1.1)	0.091	0.7 (0.2, 2.4)	0.551
	Good	25 (80.6)	6 (19.4)	0.2 (0.1, 0.7)	0.011	0.1 (0.0, 0.7)	0.019*

* is for *P*-value less than 0.05 on the multivariable level analysis, and R is for the Reference category

## Discussion

This study aimed to determine the prevalence of burnout and to identify factors associated with burnout among medical students at DBU. The study demonstrated that 34.0% of medical students at DBU had symptoms of burnout. Burnout was significantly associated with reporting being less than satisfied by practice lecturers, having poor social support, and being less than satisfied by the education system.

Our finding on the prevalence of burnout is similar with the results reported in Jimma referral hospital health professional, Ethiopia (36.7%) [26] and Pakistan medical students (30.6%) [10]. On the other hand, our finding was lower than the prevalence report among medical students in the US, India, Malaysia, and Saudi Arabia, (45 to 70%) [3–5, 11–13], while higher than the report of less than 27% in United Kingdom (University of Saint Andrews, and University of Manchester), and Brazil (Fortaleza medical school and Universidad Federal de Sergipe) [7–9]. Some of the possible reasons for these differences might be due to the difference in culture, socioeconomic status, and study population. Some of the studies only included third- and fourth-year medical students while our study included students from the first year to graduate class students. Additionally, the different in burnout assessment tool and sample size may contributed to this discrepancy. Besides medical students of DBU came from being employed to an unemployed student which may contribute to the difference in the prevalence of the burnout status.

In agreement with previous studies [3, 6, 8, 10], we found that dissatisfaction by practical lecturers, moderate social support, and satisfaction on their education significantly associated with burnout. In this study, burnout was not significantly associated with gender, years of education, stress, little control over life, lower level of physical activity, cigarette smoking, excessive alcohol drinking, lack of confidence in clinical skills, age, shortage of off-time, fear of the consequences of failure, family responsibilities, uncertain future, and increased workload. However, other studies [3, 6–10, 16] found significant association between these factors and burnout. This variation may be due to difference in study population, sample size, culture and socioeconomic status. Most of the students in our study were male and between the age of 24 and 38 years. The small sample size of our study might not allow us to explore the factors associated with burnout.

In this study, there are several areas showing dissatisfaction with the course, which might explain why burnout is more prevalent among medical students of DBU. Theoretically, burnout is defined as a measure of professional distress with three main domains: excess EE, excess DP, and reduced PA [1], all this three domains might be directly or indirectly related to satisfaction. It is found in this study that dissatisfaction with the course in medical students at DBU is the main reason for the higher prevalence of burnout at this population. The findings here may be generalizable to other areas in the world, and dissatisfaction with the course may explain why burnout is more prevalent among medical student in general.

We measured burnout using a standard tool with demonstrated reliability and validity. To our knowledge, this is the first study that assessed burnout among all class years of medical students in Ethiopia. Our findings may be generalizable to other low- and middle-income countries. Despite these strengths, this study has the following limitations. First, we noticed instability in the the multivariable model that may be related to the sample size. To optimize the model, we removed variables with a small observations from the multivariable model. Second, the cross-sectional nature of the study does not allow attribution of causality inference. Finally, the tool was not validated in our study population although we revealed excellent reliability, future validation might be required.

The results of this study would have implications for developing an intervention strategy to promote the mental health of future doctors and to maximize the benefit of medical doctors on the health care system. Additionally, this study may provide a platform to develop evidence-based interventions for students experiencing burnout. Moreover, this study will serve as a baseline for government agencies (such as Ministry of Health), non-governmental organizations, policymakers, and health planners for future planning and interventions of appropriate strategies. Finally, the findings may be helpful for medical schools to help improve their students mental health.

## Conclusions

In summary, this study reported a high prevalence of burnout among medical students of DBU, Ethiopia. Dissatisfaction with the course found to be the strong predictor of burnout. The college of medicine, Debre Berhan referral hospital, and the student clinic of DBU should undertake integrated interventions to tackle the effects of burnout on students’ academic performance. The high prevalence of burnout also suggests that immediate preventive, curative, and promotive measures to be implemented. It is believed that a significant proportion of students with burnout may be also at risk of getting depression or other mental health problems including suicide. Therefore, addressing both the mental and social well-being of future doctors needs to be a priority for the medical school administration. Furthermore, further research is recommended to validate the measurement tools for burnout in Ethiopia. Future studies are also recommended to investigate the longitudinal change in burnout symptoms and its effect on the educational attainment of students during their entire medical study period.

## Supplementary information

**Additional file 1.** Cleaned Data Used in the Analysis.

## Data Availability

The cleaned data used during this study was presented as additional file 1 with comma-delimited form (*.csv). As well the raw dataset, used during this study can be available from the corresponding author (YG) upon reasonable request.

## Abbreviations

AOR: Adjusted Odds Ratio
CI: Confidence Interval
DBU: Debre Berhan University
DP: Depersonalization
EE: Emotional Exhaustion
ETB: Ethiopian Birr
GPA: Grade Point Average
MBI: Maslach Burnout Inventory
MBI-HSS: Maslach Burnout Inventory-Human Service Survey
OR: Odds Ratio
PA: Personal Accomplishment
SD: Standard deviation
STROBE: Strengthening the Reporting of Observational Studies in Epidemiology
UK: United Kingdom
US: United State

## References

[1] Plutchik R (1983). Burnout: The Cost of Caring—by Christina Maslach, Ph. D.; Prentice-Hall, Englewood Cliffs, New Jersey, 1982**,** 192 pages, 13.95hardcover, 6.95 paperbound. Psychiatr Serv.

[2] Dyrbye LN, West CP, Satele D, Boone S, Tan L, Sloan J, Shanafelt TD (2014). Burnout among U.S. medical students, residents, and early career physicians relative to the general U.S. population. Acad Med.

[3] Dyrbye LN, Thomas MR, Huntington JL, Lawson KL, Novotny PJ, Sloan JA, Shanafelt TD (2006). Personal life events and medical student burnout: a multicenter study. Acad Med.

[4] Dyrbye LN, Thomas MR, Massie FS, Power DV, Eacker A, Harper W, Durning S, Moutier C, Szydlo DW, Novotny PJ (2008). Burnout and suicidal ideation among U.S. medical students. Ann Intern Med.

[5] West CP, Shanafelt TD, Kolars JC (2011). Quality of life, burnout, educational debt, and medical knowledge among internal medicine residents. Jama.

[6] Santen SA, Holt DB, Kemp JD, Hemphill RR (2010). Burnout in medical students: examining the prevalence and associated factors. South Med J.

[7] Cecil J, McHale C, Hart J, Laidlaw A (2014). Behaviour and burnout in medical students. Med Educ Online.

[8] Costa EF, Santos SA, Santos AT, Melo EV, Andrade TM (2012). Burnout Syndrome and associated factors among medical students: a cross-sectional study. Clinics (Sao Paulo, Brazil).

[9] Almeida GC, Souza HR, Almeida PC, Almeida BC, Almeida GH (2016). The prevalence of burnout syndrome in medical students. Arch Clin Psychiatry (São Paulo).

[10] Muzafar Y, Khan HH, Ashraf H, Hussain W, Sajid H, Tahir M, Rehman A, Sohail A, Waqas A, Ahmad W (2015). Burnout and its associated factors in medical students of Lahore**,** Pakistan. Cureus.

[11] Bera T, Biswas NM, Mandal A, Ghosh A, Bhattacharya S, Bera S (2013). Burn out among medical students-a study across three medical colleges in eastern India.

[12] Chin RWA, Chua YY, Chu MN, Mahadi NF, Yusoff MSB, Wong MS, Lee YY. Prevalence of Burnout among Universiti Sains Malaysia Medical Students. Educ Med J. 2016;8(3):61-74.

[13] Albalawi AE, Alhawiti TS, Aldahi AS, Mohammed Y, Alshehri SK, Mirghani HO (2015). The assessment of the burnout syndrome among medical students in Tabuk University, a cross-sectional analytic study.

[14] Talih F, Warakian R, Ajaltouni J, Shehab AA, Tamim H (2016). Correlates of depression and burnout among residents in a Lebanese Academic Medical Center: a cross-sectional study. Acad Psychiatry.

[15] Atalayin C, Balkis M, Tezel H, Onal B, Kayrak G (2015). The prevalence and consequences of burnout on a group of preclinical dental students. Eur J Dent.

[16] Ashkar K, Romani M, Musharrafieh U, Chaaya M (2010). Prevalence of burnout syndrome among medical residents: experience of a developing country. Postgrad Med J.

[17] Ishak W, Nikravesh R, Lederer S, Perry R, Ogunyemi D, Bernstein C (2013). Burnout in medical students: a systematic review. Clin Teach.

[18] Maslach C, Schaufeli WB, Leiter MP (2001). Job burnout. Annu Rev Psychol.

[19] Piko BF (2006). Burnout, role conflict, job satisfaction and psychosocial health among Hungarian health care staff: a questionnaire survey. Int J Nurs Stud.

[20] Kumar Shailesh (2016). Burnout and Doctors: Prevalence, Prevention and Intervention. Healthcare.

[21] Adam S, Cserhati Z, Meszaros V (2015). High Prevalence Of Burnout And Depression May Increase The Incidence Of Comorbidities Among Hungarian Nurses. Ideggyogyaszati szemle.

[22] Kitaoka-Higashiguchi K, Morikawa Y, Miura K, Sakurai M, Ishizaki M, Kido T, Naruse Y, Nakagawa H (2009). Burnout and risk factors for arteriosclerotic disease: follow-up study. J Occup Health.

[23] Arandjelovic M, Ilic I, Jovic S (2010). Burnout and the quality of life of workers in food industry--a pilot study in Serbia. Vojnosanit Pregl.

[24] Selamu M, Thornicroft G, Fekadu A, Hanlon C (2017). Conceptualisation of job-related wellbeing, stress and burnout among healthcare workers in rural Ethiopia: a qualitative study. BMC Health Serv Res.

[25] Weldegebriel Z, Ejigu Y, Weldegebreal F, Woldie M (2016). Motivation of health workers and associated factors in public hospitals of West Amhara, Northwest Ethiopia. Patient Prefer Adherence.

[26] Biksegn A, Kenfe T, Matiwos S, Eshetu G (2016). Burnout status at work among health care professionals in aTertiary hospital. Ethiop J Health Sci.

[27] Salilih SZ, Abajobir AA (2014). Work-related stress and associated factors among nurses working in public hospitals of Addis Ababa, Ethiopia: a cross-sectional study. Workplace Health Saf.

[28] Maslach C, Jackson SE (1981). The measurement of experienced burnout. J Organ Behav.

[29] Maslach C, Jackson SE, Leiter MP (1996). MBI: Maslach burnout inventory: CPP, incorporated Sunnyvale (CA).

[30] Montiel-Company JM, Subirats-Roig C, Flores-Marti P, Bellot-Arcis C, Almerich-Silla JM (2016). Validation of the Maslach burnout inventory-human services survey for estimating burnout in dental students. J Dent Educ.

[31] Dalgard OS, Dowrick C, Lehtinen V, Vazquez-Barquero JL, Casey P, Wilkinson G, Ayuso-Mateos JL, Page H, Dunn G (2006). Negative life events, social support and gender difference in depression: a multinational community survey with data from the ODIN study. Soc Psychiatry Psychiatr Epidemiol.

[32] Dalgard O (1996). Community health profile as tool for psychiatric prevention. Promot Mental Health.

[33] Nosikov A, Gudex C (2003). Development of a common instrument for mental health. EUROHIS.

